# Seasonal variation in airborne infection risk in schools due to changes in ventilation inferred from monitored carbon dioxide

**DOI:** 10.1111/ina.12818

**Published:** 2021-03-08

**Authors:** Carolanne V. M. Vouriot, Henry C. Burridge, Catherine J. Noakes, Paul F. Linden

**Affiliations:** ^1^ Department of Civil and Environmental Engineering Imperial College London London UK; ^2^ School of Civil Engineering Woodhouse Lane University of Leeds Leeds UK; ^3^ Department of Applied Mathematics and Theoretical Physics Centre for Mathematical Sciences University of Cambridge Cambridge UK

**Keywords:** airborne infection risk, COVID‐19, infection modeling, monitored CO_2_, SARS‐CoV‐2, school

## Abstract

The year 2020 has seen the world gripped by the effects of the COVID‐19 pandemic. It is not the first time, nor will it be last, that our increasingly globalized world has been significantly affected by the emergence of a new disease. In much of the Northern Hemisphere, the academic year begins in September, and for many countries, September 2020 marked the return to full schooling after some period of enforced closure due to COVID‐19. In this paper, we focus on the airborne spread of disease and investigate the likelihood of transmission in school environments. It is crucial to understand the risk airborne infection from COVID‐19 might pose to pupils, teachers, and their wider social groups. We use monitored CO_2_ data from 45 classrooms in 11 different schools from within the UK to estimate the likelihood of infection occurring within classrooms regularly attended by the same staff and pupils. We determine estimates of the number of secondary infections arising via the airborne route over pre/asymptomatic periods on a rolling basis. Results show that, assuming relatively quiet desk‐based work, the number of secondary infections is likely to remain reassuringly below unity; however, it can vary widely between classrooms of the same school even when the same ventilation system is present. Crucially, the data highlight significant variation with the seasons with January being nearly twice as risky as July. We show that such seasonal variations in risk due to changes in ventilation rates are robust and our results hold for wide variations in disease parameterizations, suggesting our results may be applied to a number of different airborne diseases.

**Practical implications**

- The methodology presented herein can be readily applied to any classroom with CO_2_ monitoring provision, leading to an estimate of the number of secondary infections for airborne transmission from a single original infector.
- It can also be used for a wider range of airborne diseases as well as a general indicator of indoor health.

## 1 INTRODUCTION

Since the outbreak began in December 2019, the novel coronavirus disease (COVID‐19) caused by the SARS‐CoV‐2 virus has spread around the world resulting in a global pandemic that has become the predominant feature of 2020–2021. In an effort to contain the spread of the virus, the UK, along with many other nations, implemented lockdown measures including a “stay at home” order from March 2020, causing most public spaces to close. This led to schools closing, for all but children of key workers and children within certain vulnerable groups, resulting in most pupils learning from home, with only some receiving remote provision. With the new academic year 2020–2021 and the ongoing attempts to rekindle the economy, UK schools reopened in September 2020. The occupancy of the same spaces by staff and students over long durations throughout the working week makes it a crucial space in which to determine the risk that COVID‐19 might present to the millions of pupils and, through transmission, their families and social groups, around the country. It is especially important to understand how this risk might vary under different conditions, notably changes in the seasons and the approach of winter, where time spent indoors can be expected to increase. Although children seem to be less likely to become seriously ill from COVID‐19, it is known that they can still get infected^1^ and so increase the chance of spreading the virus to more susceptible members of the population, including teachers and elderly relatives.

Most COVID‐19 transmissions are thought to happen indoors (as shown, e.g. by Quian et al.,^2^ with the analysis of hundreds of outbreaks in China) through three routes: the droplet (or spray) route, the contact (or touch) route, and the airborne (or aerosol) route. These, along with their respective mitigation strategies, are detailed and discussed in a number of papers including.^3^ We focus herein on relatively far‐field (>2 m) airborne transmission where small infected respiratory droplets and aerosols remain suspended in the air where they can be transported by indoor air currents and inhaled. It is thought that this route may be significant in the spread of COVID‐19, with growing evidence showing that it played a role in well‐documented outbreaks: for example in the case of the Skagit Valley Chorale event for which Miller et al.^4^ show that aerosol transmission has to be considered in order to account for the extensive resulting number of cases. While measures such as social distancing and appropriate cleaning procedures will reduce the risk linked to the droplet and contact routes, infections due to airborne transmission may be harder to contain. They require, along with other measures,^5^ the appropriate use of ventilation as recommended by the WHO^6^ but, as described by Bhagat et al.,^7^ the physics of indoor flows are complex, and thus, the consistent provision of clean air at appropriate rates to all desired locations within indoor spaces remains a scientific and engineering challenge.

To analyze the likelihood of airborne infection from COVID‐19 caused by attendance at schools, this paper adopts the approach described in the seminal works of Riley et al.^8^ and Rudnick & Milton,^9^ which were successfully applied to the transmission of measles, rhinovirus, and influenza.^10^ We predict the absolute risk of airborne infection over a period of time in a given space, by taking CO_2_ measurements and occupancy profiles and calculating the number of secondary infections, or the number of infections due to a single originally infected individual. We assume, as is currently required, that anyone showing any symptoms ceases attending school. We further assume that staff and students spend the vast majority of their school day in the classroom and thus infer that the risk of airborne infection is dominated by their time in the classroom which is currently likely to be the case in most schools, with break times being taken outdoors (minimal airborne infection risk) or within the classroom in inclement weather. We therefore calculate the likely consequences of airborne infection, the number of secondary infections, for pre/asymptomatic periods where the original infector continues to regularly attend the space, which, in this instance, is a classroom. The pre/asymptomatic period may be especially significant, as it corresponds to the period where infectivity is thought to reach a peak.^11^

This approach is particularly applicable to primary schools (5–11 years old) where the same group of students can be assumed to attend the same classroom every day. It also remains suitable for secondary schools in which strategies have been implemented to reduce mixing between students, introducing, for example, fixed bubbles or groups of students in one classroom.

In addition, this methodology is useful because it does not require measurements nor estimates of ventilation flow rates, which are seldom recorded. Instead, it relies on the use of monitored CO_2_ which is becoming increasingly widespread in newly built schools in the UK in an effort to assess the performance of ventilation systems along with showing compliance to regulations (e.g. the Department for Education^12^ guidance). The focus on airborne transmission makes this study relevant to a wider range of airborne diseases, such as measles, influenza, or SARS, their spread being closely linked to ventilation as evidenced by Li et al.^13^ Finally, this work provides a tool to assess Indoor Air Quality in schools more generally and thus ensure that pupils are provided with a suitable healthy learning environment.

We describe the application of the methodology to monitored CO_2_ data in section 2 and how a measure of virus emission, the quanta generation rate, was chosen in section 3. Section 4 shows how the infection risk is estimated for a range of UK schools and how the resulting number of secondary infections varies between classroom and with the seasons. Finally, we draw conclusions in section 5.

## 2 LIKELIHOOD OF AIRBORNE INFECTION FOR CLASSROOMS AND NUMBER OF SECONDARY INFECTIONS ARISING VIA THE AIRBORNE ROUTE

Focusing only on transmission via the airborne route, we wish to determine the likely number of secondary infections that might arise within a given space should an infected individual attend the space. We assume here that all infections originate from a single infected individual. For many indoor spaces, a wide variety of people come and go with varying frequency, which makes the prediction of risk challenging. However, other indoor spaces are attended on a regular basis, day‐in‐day‐out, and for significant durations each day by the same (or similar) group of people. We term these spaces “regularly attended spaces.” Examples of these spaces include open‐plan offices and school classrooms, the latter being the focus of this study.

In order to determine the likely number of secondary infections, S_I_, from a classroom we must first calculate the likelihood that airborne infection occurs when an infected individual regularly attends the classroom for some duration. This duration is often arbitrary, and a number of reasonable choices could be made, each leading to a different likelihood. However, for regularly attended spaces, for example, a school classroom, it is reasonable to assume that once an infected individual exhibits symptoms they cease attending. Thus, one can assess the likelihood of infection occurring during the pre/ asymptomatic infectious period, assuming the infector ceases to attended once symptoms develop. For COVID‐19, the pre/ asymptomatic infectious period is estimated to be 5–7 days, therefore allowing us to examine the likelihood of infection occurring over five consecutive working days; that is, a duration *T_A_* = 5 weekdays.

For infection to occur via the airborne route, a susceptible occupant of the classroom must breathe in infectious particles that are being carried on the indoor air currents. The infectious particles, or aerosols, originate in the exhaled breath of an infector, and we assume that they remain in the “rebreathed air,” that is, air that has already been breathed by another individual, which we take as a suitable surrogate for estimating airborne infection risk. The resulting probability of infection *P_A_* was described by Riley et al.^8^ and then extended by Rudnick & Milton,^9^ as

(1) $$P_{A}=1‐\exp\left(‐\int_{0}^{T_{A}}\lambda \text{dt}\right)=1‐\exp\left(‐\frac{I}{n}q\int_{0}^{T_{A}}f\;\text{dt}\right),$$

where *λ* is the infectivity rate, *I* is the number of infectors, *n* is the number of occupants in the space, *q* is the emission rate of infectious doses, known as the quanta generation rate (see section 3), and *f* is the fraction of rebreathed air. This, in turn, is defined as *f* = (*C*−*C*_0_)/*C*_a_, with *C* the monitored CO_2_ within the space, *C*_0_ indicating the outdoor ambient level and *C_a_* the concentration of CO_2_ added through exhaled breath. This assumes that the main source of CO_2_ is occupants, which is pertinent in classrooms and other spaces without combustion sources. In both the original formulation of Riley et al.^8^ and latter form of Rudnick & Milton,^9^ Wells‐Riley models calculate the complement of the probability that no‐one becomes infected within the duration (in our case *T__A_*; hence, the exponential terms in (1) are by no means representative of the response to a cumulative dose. The method presented here assumes implicitly that susceptible people attending the classroom will not become infected elsewhere or by another route than the airborne one. This assumption allows us to determine the contribution of a specific setting (in our case the classroom) and transmission route (in our case airborne) to the spread of the disease, and thus quantify how it might vary with environmental factors as the seasons change.

Equation (1) cannot be directly applied to durations which cover varied occupancy, such as a classroom with students coming in during the day and empty at night. Herein, we assumed that each classroom was regularly attended by *N* = 33 people (typical in UK state schools) and the occupancy *n* varied between zero and *N* according to the school timetable (which was typically accessed through online records). The number of infectious individuals *I* was set to be a constant fraction of the current occupants; that is, *I* = *n*/*N*, which led to *I/n* = 1 when the space was fully occupied and *I* = 0 when empty. This rendered the fraction of occupants infected constant as *I*/*n* = 1=*N*. With these assumptions, the likelihood *P_A_* of airborne infection occurring due to a single infectious person attending school during a pre/asymptomatic period can then be calculated using:

(2) $$P_{A}=1‐\exp\left(‐\frac{1}{N}\int_{0}^{T_{A}}\sigma (t)f\;q\;\text{dt}\right),$$

where the term σ(*t*) is a Heaviside operator based on whether the space is occupied or unoccupied, defined by

$$\sigma \left(t\right)=\left\{\begin{array}{c} 1,\;\text{if}\;\text{the}\;\text{room}\;\text{is}\;\text{occupied}\;(n\neq 0),\\ 0,\;\text{otherwise} \end{array}\right)$$

This likelihood then provides the expected number of secondary infections that might arise via the airborne route from an infectious pre/asymptomatic person regularly attending school, via

(3) $$S_{I}=\left(N‐1\right)P_{A}$$

The rebreathed fraction *f* was found from data sets of CO_2_ levels monitored in schools, described in section 4, where we took *C*_0_ to be the average CO_2_ within the space between the hours of 05:00 and 06:00 each day (which corresponds to an unoccupied space which has had time to reach a baseline CO_2_ level after occupation of the previous day) and *C*_a_ = 37,500 ppm.^9^ The choice of a suitable value for the quanta generation rate *q* is discussed in section 3. We note that no assumption as to the distribution of CO_2_ within the classroom has been made in deriving (2). As such, (2) provides the probability of airborne infection occurring at the sensor location, under the above assumptions, with the additional assumption that infectious airborne material is uniformly mixed but only within the rebreathed air. Hence, in order to employ (2) it is not required to assume that all of the air within the classroom is well‐mixed but it is required to assume that the infected breath is evenly mixed within the rebreathed air.

Highlighting this emphasizes that when multiple CO_2_ sensors are present within a single indoor space then differing CO_2_ levels recorded by those sensors are not inherently contradictory and may indicate that variation of risk within the space. CO_2_ variations within an indoor space such as a classroom and the related judicious placement of monitoring sensors remain a challenging problem worthy of further investigation. In most settings, CO_2_ measurements must remain unobtrusive, and thus, placing sensors on walls is a sensible choice. This means that direct measurements of the exhaled breath are avoided along with their complications, detailed, for example, by Melikov & Kaczmarczyk^14^ and Kierat et al.^15^ Sensors should ideally placed far away from windows or other openings, and, crucially to study infection risk, they should also remain within the height where occupants breathe in. In this study, our data come from classrooms within each of which a single CO_2_ sensor was placed at a height within the breathing zone, which was deemed suitable to ensure ventilation control. The impact of the exact location of the sensor is further increased by the choice of a constant and uniform concentration of CO_2_ and the calculation of secondary infections. Indeed, when estimating the number of secondary infections, via (3), one is assuming the airborne infection risk is appropriate for all susceptible occupants; an assumption which is valid in the case that all the air within the space is well‐mixed.

Both *P_A_* and *S_I_* were calculated for all 5 weekday periods within the data gathered on a rolling basis (a total of 15,000 pre/asymptomatic periods in total). In the rolling absolute number of secondary infections reported herein, we exclude all values for which the 5 weekday period contained any unoccupied days, for example, those that included weekdays that fell in the school holidays.

The use of the measurement of CO_2_ to infer the risk of airborne infection introduces several uncertainties caused by the choice of sensor location and the sensor itself. The sensors used in this study have been installed to control the ventilation provision, their location is fixed throughout the measuring period, and it can be assumed that they have been designed and maintained appropriately. In addition, in order to limit the impact of these uncertainties as well as the ones introduced by a choice of quanta generation rate (see section 3), this paper focuses on reporting relative risks rather than absolute numbers.

### 2.1 Determining appropriate quanta generation rates for school classrooms

For all infection risk modeling based on the Wells‐Riley approach, the quanta generation rate, *q*, is invariably the hardest input parameter to quantify and the resulting uncertainty is typically a dominant factor, as it varies with disease, individuals, and occupant activity levels.^10^, ^16^ Given this uncertainty, we discuss the changes in our results that would arise from other feasible choices of the quanta generation rate. Estimates of quanta generation rates have been made via a variety of methods for a host of airborne diseases. Understandably, for SARS‐CoV‐2, choices are limited but we apply the values reported in the work of Buonanno et al.^17^ who consider the likely values of *q* based on viral load and respiratory activity. As a value which we deem to be typically appropriate for school classrooms, we take a value of *q* = 1 quanta/h—this is obtained by taking *c_x_* = *c_i_c_v_* ≈ 7 x 10^6^ RNA/ml, where *c_i_* = {0.1,0.01} is the ratio between infectious quantum and the infectious dose expressed in viral RNA copies, and *c_v_* = {7 x 10^7^, 7 x 10^8^} RNA/ml is the viral load measured in sputum. These values assume that, for most of the time within school classrooms, the students are sitting breathing with perhaps a small number vocalizing: the data for whispered counting fall between these two activities and are closer to breathing. As such, for our base case, we take data for whispered counting from Buonanno et al.^17^ and use their results to map our selected values of *c_x_* to values of quanta generation rates *q*. We note that all of the work underlying the results of Buonanno et al.^17^ concerned adults and, for COVID‐19, it is not known if appropriate values for the quanta generation rates differ significantly between adults and children. The quanta generation rate might also be expected to vary for a single individual during the course of the infection, but once again, information is limited in the case of SARS‐CoV‐2 and we take *q* to be constant in time.

Throughout our results, we comment on the changes that would arise if the students within the classroom were (on average) all vocalizing/talking (eg, a noisy classroom), taking again *c_x_* ≈ 7 x 10^6^ RNA/ml gives *q* ≈ 5 quanta/h. For our key findings, that is, the seasonal variation in airborne infection risk, we also comment on the results of assuming much higher quanta generation rates of *q* ≈ 20 quanta/h and *q* ≈ 100 quanta/h which may represent higher viral loads or higher levels of activity.

## 3 RESULTS FOR THE SEASONAL VARIATION IN AIRBORNE INFECTION IN SCHOOLS

The data presented originate from recently built or renovated school classrooms, since these are more likely to have existing CO_2_ monitoring provision installed. Overall, 45 spaces are monitored from within 11 different schools (8 primary and 3 secondary) and span the period November 2015 to March 2020 (when schools were subject to a UK wide lockdown), as detailed in Table 1. The data originate from schools in England, as far north as Yorkshire, as far south and west as Somerset, and as far east as Kent; the data are sourced from schools in a mix of urban and rural settings. The ventilation system varied between classrooms but was, in all cases, a hybrid ventilation system which switched between naturally driven ventilation and mechanically driven ventilation modes depending on the conditions. The ventilation provision was controlled by automatic operation of louvers, vents, and fans. These classrooms were also typically fitted with additional windows or vents that could be opened manually by the classroom occupants.

**TABLE 1:** Schools where the monitored CO_2_ data originate from

School	Type	County	Rooms	Data span
1	Primary	Yorkshire	22	Nov/15–Mar/19
2	Secondary	Berkshire	1	Nov/19–Mar/20
3	Primary	Somerset	1	May/17–Mar/18
4	Primary	Surrey	1	Dec/17–May/18
5	Primary	Cambridgeshire	2	Aug/17–Jan/18
6	Primary	Not disclosed	3	Dec/18–Feb/19
7	Primary	Essex	4	Oct/16–Dec/17
8	Secondary	Kent	1	Mar/18–Apr/19
9	Primary	Surrey	4	Aug/17–Aug/18
10	Primary	Kent	1	Aug/17–Jul/18
11	Secondary	Hertfordshire	5	Sep/18–Mar/20

### 3.1 The airborne infection risk in one classroom in January and in July

**FIGURE 1:**
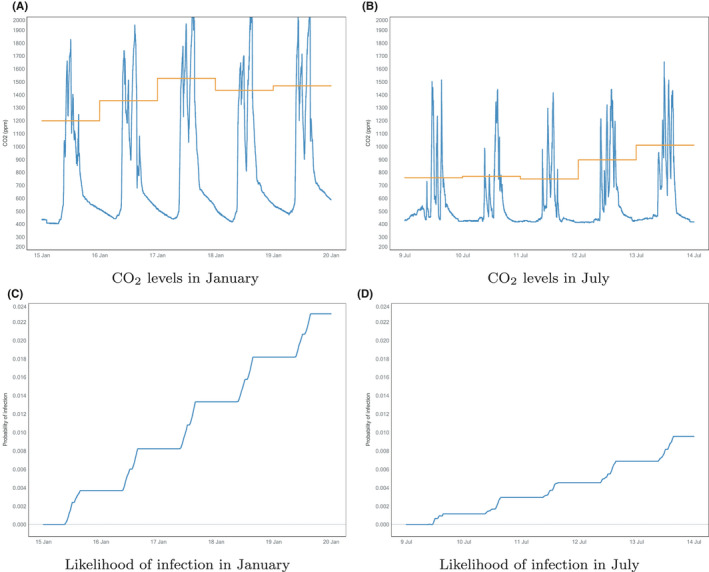
The variation in CO_2_, top panes (A and B), over a pre/asymptomatic period within classroom Y1‐2 and, bottom panes (C and D), the corresponding probability of infection (assuming *q* = 1 quanta/h). The orange horizontal lines in A and B show the daily averaged CO_2_ during the occupied periods (defined for school classrooms as 09:00 and 16:00, see^12^). The left‐hand panes (A and C) show data for a pre/asymptomatic period in January 2018; the right‐hand panes, (B and D), show data for a period in July 2018

To determine the airborne infection risk in schools, we begin by taking one classroom (selected at random) from within our set (45 classrooms in total). For the year 2018, we plot the variation in CO_2_ level in the upper panes of Figure 1 for two periods (5 school days) one in January and one in July. The data from January show that, in adherence with the Department for Education^12^ guidance for a naturally ventilated classroom, the daily average CO_2_ level is kept below around 1500 ppm, and the CO_2_ levels only spike above 2000 ppm for very short periods. Moreover, this was the case for all classrooms that we examined throughout almost all the periods we had data (spanning November 2015 to March 2020), and hence enables us to make some comment on whether adherence to the existing guidance might provide adequate airborne infection risk for COVID‐19.

What is stark from Figure 1 is the decrease in CO_2_ levels, both in terms of daily mean and in terms of peak values, in July compared with January. It can be inferred from the gradual drop toward ambient CO_2_ levels during January nights that this classroom can be relatively well‐sealed when thermal conditions require and there is no need for ventilation. Compare this with July when, presumably, warmer outdoor conditions did not require all openings to be shut at the end of the day and CO_2_ levels attain ambient levels for most of the night. Daytime excess CO_2_ levels in July typically attain values around half those in January despite occupancy and activity levels which are not expected to vary significantly. This is clear evidence of increased ventilation levels in July in response to warmer weather that are either provided automatically by the ventilation system and/or by occupant intervention, for example, opening a window. In any event, the change in risk is significant with the likelihood of airborne infection of COVID‐19 being more than twice as high in January compared with July. As expected, in both cases the risk increases only during occupied hours producing a “staircase” of increase likelihood over the pre/ asymptomatic period. Accounting for an accuracy of ±50 ppm in the CO_2_ measurements leads to an uncertainty of ±0.0012 in the predicted likelihood of infection after 5 working days shown in Figure 1. We note that the absolute probabilities of airborne infection remain low in both cases, implying that changes in the likelihood will exhibit an approximately linear response to changes in the underlying parameters, for example, the quanta generation rate, and that results for the relative risk will be robust while the probabilities remain low.

### 3.2 The variation in airborne infection risk within one school

**FIGURE 2:**
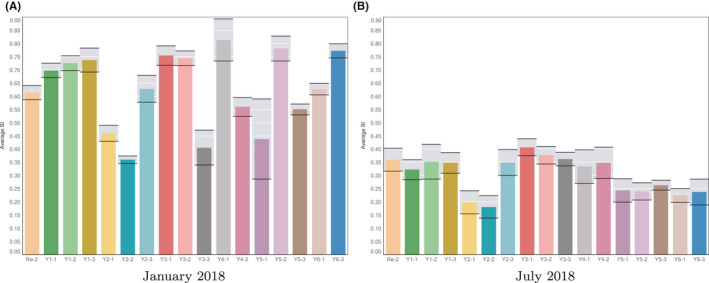
Variation in average number of secondary infections (*S_I_*) between classrooms of a primary school for the most and least risky months (January and July, respectively) in the school year 2017–2018

Figure 2 shows the average number of secondary infections for 17 different classrooms within a typical primary school over January 2018 and July 2018, respectively. These are detailed in Table 2 and were chosen because they allowed a direct comparison of similar spaces within the same school for the months under study. What is apparent from the figure is that, as one would expect, the number of airborne secondary infections in winter (January) greatly exceed those of summer (July). For the classrooms shown the average *S_I_* in January is 0.63, falling to 0.30 in the July of that year; this is assuming a quanta generation rate of *q* = 1 quanta/h and we return to a discussion of these values in section 4.3. The change in risk between January and July is not easily classified; many of the relatively risky classrooms in January remain so in July (eg, Re‐2, Y1‐1, Y1‐2, Y1‐3, Y3‐1, and Y3‐2), and some of the relatively low‐risk classrooms in January remain so in July (eg, Y2‐1, Y2‐2, Y3‐3, and Y5‐1), but others change from being relatively risky to relatively low risk (Y4‐1, Y5‐2, and Y6‐3) or from being relatively low risk to being relatively risky (Y3‐3). Interestingly, we found no obvious correlation between these differences and the location of the classroom in the school, for example, whether located on the ground or first floor, or the proximity of one classroom to another.

**TABLE 2:** Description of the 17 primary school classrooms considered

Classroom	Elevation	Floor area (m^2^)	Year	Age group
Re‐2	Ground floor	67	Reception	4–5
Y1‐1	Ground floor	66	Year 1	5–6
Y1‐2	Ground floor	67	Year 1	5–6
Y1‐3	Ground floor	66	Year 1	5–6
Y2‐1	Ground floor	67	Year 2	6–7
Y2‐2	Ground floor	67	Year 2	6–7
Y2‐3	Ground floor	66	Year 2	6–7
Y3‐1	First floor	60	Year 3	7–8
Y3‐2	First floor	60	Year 3	7–8
Y3‐3	First floor	60	Year 3	7–8
Y4‐1	First floor	60	Year 4	8–9
Y4‐2	First floor	60	Year 4	8–9
Y5‐1	First floor	60	Year 5	9–10
Y5‐2	First floor	61	Year 5	9–10
Y5‐3	First floor	60	Year 5	9–10
Y6‐1	First floor	60	Year 6	10–11
Y6‐2	First floor	60	Year 6	10–11

What is also noticeable is the relatively small variation in *S_I_* within any given class within the same month. We choose to quantify this by the coefficient of variation (calculated as the ratio of the unbiased estimate of the standard deviation relative to the mean) which are, on average, 8% in January 2018 and 14% in July 2018 for the classrooms shown (see the gray bars in Figure 2). However, the variation between classrooms even within the same month is significant. The coefficient of variation of *S_I_* for the classrooms shown is 21% in January and 26% in July, with the most risky classroom being as least twice as risky as the least in both January and in July, in spite of the fact that all 17 classrooms from this particular school have the same ventilation system installed by the same contractor.

Finally, we note that variations are also observed between school years: a risky classroom one year being relatively less risky in the following academic year for instance. This could be explained by changes in the ventilation system, with adjustments being made to the building management system or interventions from the occupants, or could be due to a difference use of the space in a different academic year, for example, a class with either more or less pupils or different activities, or could be the result of differing weather conditions.

### 3.3 The seasonal variation in predicted airborne secondary infections in schools

**FIGURE 3:**
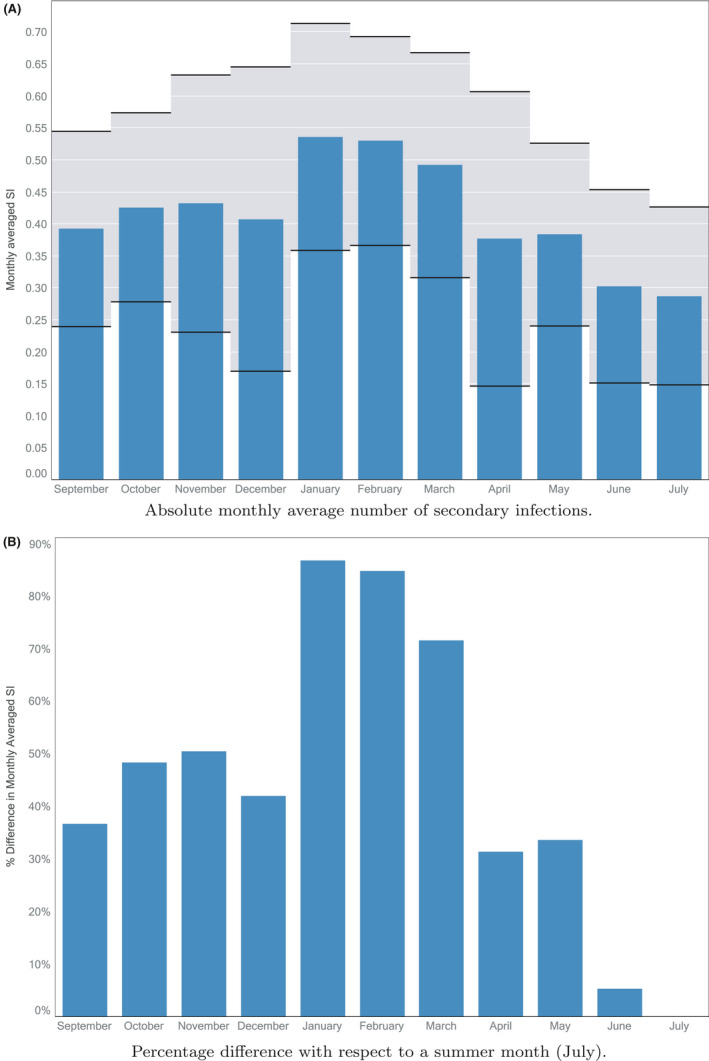
The variation in (A) the absolute, and (B) the relative, average monthly number of secondary infections across the seasons. Data shown represent CO_2_ monitoring in 45 classrooms (within 11 different schools) and spans the period November 2015 to March 2020 (see Table 1)

The variation in the absolute number of predicted secondary infections with season is presented in Figure 3A. For each of the 45 classrooms, the rolling absolute *S_I_* was calculated for all available data and values pertaining to each calendar month were then averaged (August is not represented as it falls during the school holidays). As can be seen, at the start of each academic year (September) the risk of airborne infection rises as the UK enters winter weather, with the risk peaking in January and February, after which the risk gradually subsides as more temperate spring and summer weather is approached. Taking a value for the quanta generation rate of *q* = 1 quanta/h^17^ yields the values for the absolute airborne number of secondary infections shown in Figure 3A. These lie reassuringly below unity (with a mean of *S_I_* = 0.41) and may be representative for the airborne spread of COVID‐19 in relatively quiet classrooms. However, classrooms in which children are expected to be more vocal or active (corresponding to *q* = 5 quanta/h) then one could expect the airborne *S_I_* to increase by a factor of approximately five; that is, *S_I_* ≈ 2.0. There is uncertainty as to appropriate values for the quanta generation rate for COVID‐19, with current evidence suggesting that a relatively low number of high spreading events make an unusually important contribution to the disease spread.^18^ Consequently, these absolute risk results should be treated with caution.

Before examining the seasonal variation of the relative risk, it is worth considering the variations in risk within each month. The average coefficient of variation of *S_I_* in all 45 classrooms within each single month is approximately 40%. This is approximately twice as large as the coefficient of variation for *S_I_* within each single month from a single school with 17 classrooms suggesting that, in addition to the variations intra school (see section 4.2), significant variations also exist between the different schools. We note also that all the schools had broadly similar ventilation strategies (ie, hybrid ventilation systems) and demonstrated adherence to existing ventilation guidance. Should schools with very different ventilation provision be included then one might expect wider variations: mechanically ventilated school classrooms are set more demanding ventilation provision within the guidance, while schools with uncontrolled ventilation are likely to vary more widely due to the greater influence of occupants interventions.

The relative number of airborne secondary infections is presented in Figure 3B, in which the data from Figure 3A are shown normalized by the risk in summer (here taken to be July). As the academic year begins, we see that average *S_I_* in classrooms might be expected to be about 30%–40% above the levels that would be seen in summer. Broadly speaking, levels then steadily increase toward the Christmas holidays and in January and February, average *S_I_* peak at 80%–90% above July levels. Levels remain high in March and then fall sharply back toward summer levels. This seasonal pattern in *S_I_* is consistent with intermediate ventilation provision in autumn and spring weather, highest ventilation in summer, and minimum ventilation in winter to avoid heat loss.

We tested the seasonal variation in number of airborne secondary infections in schools for varied levels of quanta generations rate. For quanta generation rates 0.1 ≤ *q* ≤ 5 quanta/h, the quantitative predictions illustrated in Figure 3B remain almost unchanged. For much high quanta generation rates 20 ≤ *q* ≤ 100 quanta/h, the qualitative trends in the data remain but the precise values change somewhat, for example, for *q* = 100 quanta/h *S_I_* in January would be predicted to be 41% higher than the July value.

## 4 CONCLUSION

Based on CO_2_ monitored within 45 classrooms from across England, we have shown that airborne infection risks in schools are likely to vary significantly with the season. Purely based on changes in environmental conditions, the expected levels of secondary infections in winter (eg January and February) are nearly double those in summer (eg July). We suggest that, while our results for schools with relatively recently installed ventilation provision indicate airborne infection risk for COVID‐19 may be low, ventilation provision for wintertime should be assessed or reviewed. It is important to note that the analysis in this paper is based on historical CO_2_ data measured before significant restrictions were introduced in the UK to control COVID‐19 transmission. The study therefore models the likely risk under normal operating conditions for the monitored schools. The UK Department for Education has issued guidance to schools recommending additional ventilation, and hence, it is likely that airborne risks over autumn 2020/winter 2021 may be lower than those predicted here. We have also shown that airborne infection risk can vary widely within a school, even in the case that the same ventilation system is installed by the same contractor. Where these differences might increase airborne infection risk, occupants should be encouraged to review their behaviors to ensure that these are appropriate. These differences may be even more exaggerated in schools with no controlled ventilation provision, which represents a significant portion of the existing school stock within the UK. Moreover, our assessment of the airborne infection risk as low may not apply to these schools but monitoring of CO_2_ would provide the evidence required to make an assessment.

The seasonal variations in airborne infection risk described in this study account only for those due to changes in the indoor environment which arise due to ventilation and/or occupants’ behaviors due to varying outdoor temperatures during the year. It is likely that the virus and the human response to it will also exhibit some seasonal variations and, as such, the risks of airborne infection might be compounded. If these increase the risk of transmission, for example, due to a weaker immune response in winter shown by Dopico et al.^19^ or to changes in humidity as described for example by Marr et al.^20^ for influenza, our results would in effect be a lower bound for seasonal variation in airborne infection risk. In addition, the risk presented herein refers to the airborne route of transmission only. The two other major disease transmission routes might also be shown to exhibit variations in infection risk, which would contribute to observed changes in total number of infections throughout the year.

Finally, the method we present is applicable to all airborne diseases. We have shown that, in particular, the variations in relative airborne infection risk with ventilation conditions, and hence season, hold over a wide range of quanta generation rates and so should be directly applicable to a wide range of diseases.

## CONFLICT OF INTEREST

The authors declare no competing interests.

## AUTHOR CONTRIBUTIONS

CVMV led the study, analyzed the data, created the figures, and wrote parts of the manuscript. HCB conceived the study, secured the data, and wrote parts of the manuscript. CJN and PFL oversaw the study, supplied guidance, and advice throughout and edited the manuscript.

### PEER REVIEW

The peer review history for this article is available at https://publons.com/publon/10.1111/ina.12818.
